# Supplementary material for: Caffeine Consumption in Switzerland: Results from the First National Nutrition Survey MenuCH
Source: Nutrients. 2019 Dec 20;12(1):28. doi: 10.3390/nu12010028 (PMC7019981; doi:10.3390/nu12010028)
Supplement: Supplementary file 1 [file nutrients-12-00028-s001.pdf]

Supplementary Files

Caffeine consumption in Switzerland: results from the first national nutrition survey menuCH

Supplementary Figure S1. Decision tree regarding food groups with or without caffeine.

Supplementary Table S1. Overview of caffeine concentration in foods, by food groups.

Supplementary Table S2. Measurements of caffeine concentration in coffee and soft drinks.

Supplementary Table S3. Daily caffeine consumption per kg of body weight.

Supplementary Figure S2. Main food groups sources of caffeine in mg/day.

Supplementary Figure S3. Distribution of caffeine consumption per hour during the day.

Supplementary Table S4. Daily caffeine consumption in the population aged 18-64 years old.

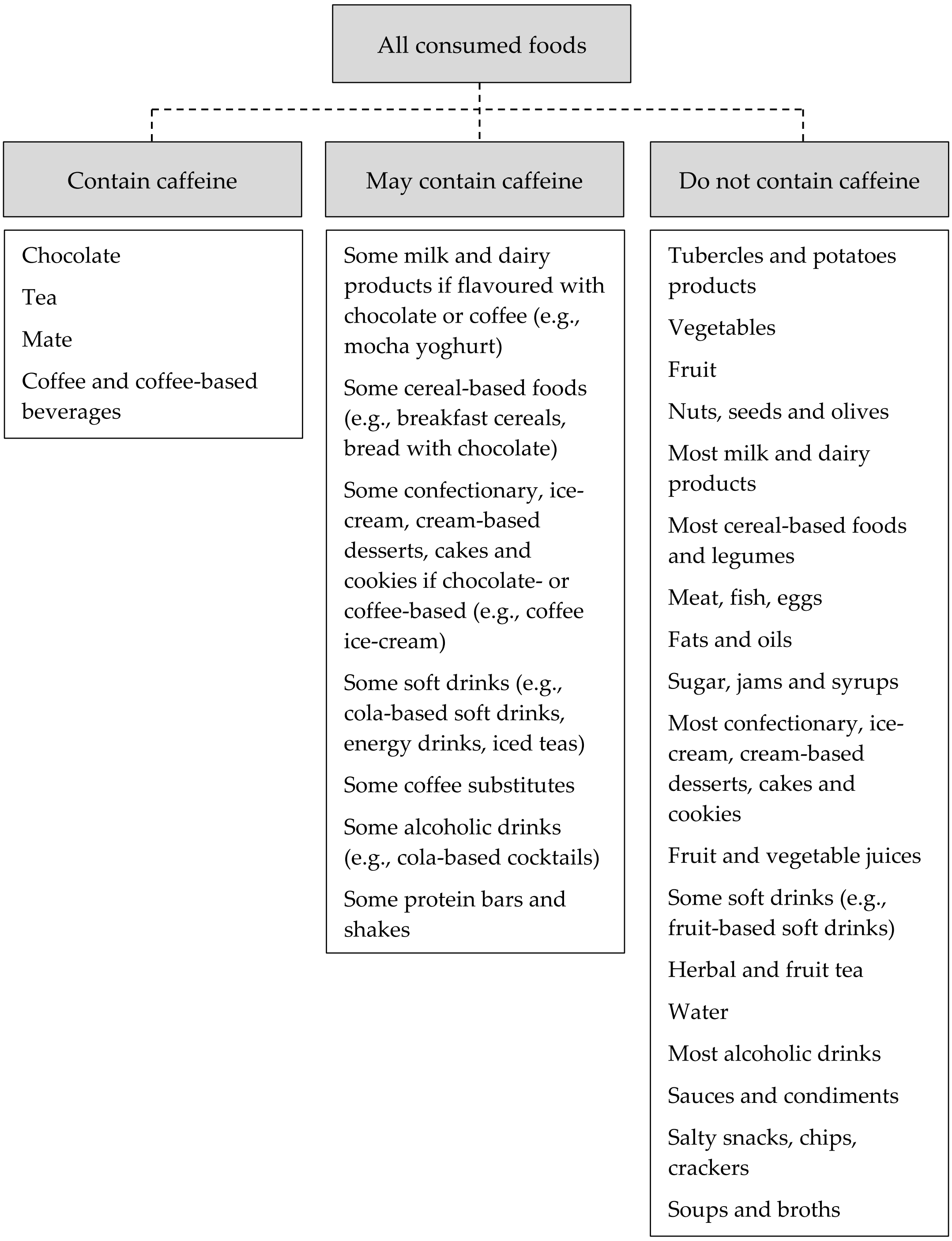

Supplementary Figure S1. Decision tree regarding food groups with or without caffeine.

Supplementary Table S1. Overview of caffeine concentration in foods, by food groups.

Food Group	Decision	Contain caffeine
**Tubercles and potatoes products**	Do not contain caffeine.	No
**Vegetables**	Do not contain caffeine.	No
**Fruit**	Even though the seeds and nuts of some fruits such as coffee, cocoa, cola and guarana contain caffeine, fruit pulp as generally consumed does not contain caffeine.	No
**Nuts, seeds and olives**	Cocoa seeds, coffee seeds, kola nuts and guarana seeds are not consumed per se, so this food group does not contain caffeine.	No
**Milk and dairy products**	Milk and dairy products do not naturally contain caffeine, but some may contain caffeine according to their flavour (chocolate, dark chocolate, milk chocolate, stracciatella, coffee/mocha). Concentrations were estimated using standard recipes, standard compositions and reference values from the American and Canadian food composition databases.	Maybe
**Cereals and legumes** *Flour, pasta, rice, bread, rusks, cereals, breakfast cereals, legumes*	Cereal-based foods and legumes do not naturally contain caffeine, but some may contain caffeine according to their flavour (chocolate, dark chocolate, milk chocolate). In addition, some breakfast cereals contain caffeine. Concentrations were estimated using standard compositions and reference values from the American and Canadian food composition databases. However, if cocoa powder or guarana extract was at the bottom of the list of ingredients, the caffeine concentration was considered as negligible.	Maybe
**Meat, meat products and meat substitutes**	Do not contain caffeine.	No
**Fish and seafood**	Do not contain caffeine.	No
**Eggs**	Do not contain caffeine.	No
**Fats and oils**	Do not contain caffeine.	No
**Sugar, jam, honey, and syrups**	Do not contain caffeine.	No
**Chocolate (pure)**	Chocolate is a source of caffeine. The American and Canadian food composition databases served as the main references: chocolate milk (20 mg/100g), dark chocolate (70 mg/100g), chocolate (average milk chocolate and dark chocolate: 45 mg/100g), and white chocolate (0 mg/100g).	Yes
**Confectionery, ice-cream, cream-based desserts, cakes and cookies**	Some products may contain caffeine according to their flavour (chocolate, dark chocolate, milk chocolate, stracciatella, coffee/mocha). The American and Canadian food composition databases served as the main references and, when the food item was missing as a reference, we estimated caffeine concentration based on standard recipes and compositions: cocoa powder (150 mg/100g), chocolate powder (30 mg/100g), chocolate milk (20 mg/100g), dark chocolate (70 mg/100g), chocolate (45 mg/100g). If the quantity/proportion of chocolate contained in the foods was not available in the ingredient lists, we estimated it. Values for foods with coffee flavour were estimated from several unlisted references, as no information was found on the caffeine concentration in the coffee extract.	Maybe

Food Group	Decision	Contain caffeine
**Fruit and vegetable juices**	Do not contain caffeine.	No
**Soft drinks**	Cola-based soft drinks, energy drinks and iced teas contain caffeine. The concentration of caffeine in the Swiss regular Coca Cola® (7 mg/100g), Coca Cola Diet® (5.8 mg/100g), Coca Cola Zero® (7.8 mg/100g), Pepsi® (7.7 mg/100g) and Pepsi Max® (10 mg/100g), and iced teas (3.6 mg/100g) were measured. Red Bull® and Energy Drink® had both the concentration on the packaging (32 mg/100g). Other soft drinks (e.g., lemonades, fruit-based soft drinks) do not contain caffeine.	Maybe
**Herbal and fruit tea**	Do not contain caffeine.	No
**Water**	Do not contain caffeine.	No
**Tea and mate**	Tea leaves and mate are sources of caffeine. Based on the American food composition database, a scientific article and other references (see table notes),the caffeine concentration was defined as follows: black tea (15 mg/100g), green tea (12 mg/100g), white tea (17 mg/100g), Jasmin tea (11 mg/100g), and mate (36 mg/100g).	Yes
**Coffee, coffee-based beverages, and coffee substitutes**	The caffeine concentration of 42 samples of ristrettos, espressos and lungos were analysed. The concentration of caffeine in "self-made" and "take-away/restaurant/vending machine" ristrettos was 254 and 268 mg/100g ("powder-based" (instant) ristrettos not measured). The concentration in "powder-based", "self-made" and "take-away/restaurant/ vending machine" espressos was 39, 91 and 177 mg/100g, respectively. The concentration in "powder-based", "self-made" and "take-away/restaurant/vending machine" lungos was 41, 51 and 101 mg/100g, respectively. The average ristretto (259 mg/100g), espressos (120 mg/100g), and lungos (67 mg/100g), and were used as a reference to estimate coffee-based beverages (e.g., cappuccino, latte macchiato) using standard recipes. Based on the American food composition database, caffeine concentration in coffee substitutes were as follows: cereal coffee (0 mg/100g), chicory coffee (2 mg/100g), mix of coffee and chicory (e.g., 40 mg/100g).	Yes
**Alcoholic drinks**	Alcohol itself does not contain caffeine, but cocktails made with cola-based soft drinks contain caffeine. In this case, the proportion of cola in the cocktail was estimated.	Maybe
**Sauces and condiments**	Do not contain caffeine.	No
**Salty snacks, chips, crackers**	Do not contain caffeine.	No
**Soups and broths**	Do not contain caffeine.	No
**Others**	Energy/sports and protein bars, and protein shakes may contain caffeine according to the ingredients and flavour. The American and Canadian food composition databases served as the main references and, when the food item was missing as a reference, we estimated caffeine concentration based on standard compositions.	Maybe

***American and Canadian food composition databases**: www.ndb.nal.usda.gov and www.food-nutrition.canada.ca. **Other references**: Sereshti H, Samadi S. A rapid and simple determination of caffeine in teas, coffees and eight beverages. Food Chemistry. 2014 Sep;158:8–13, www.caffeineinformer.com, www.frc.ch/yaourt-a-la-cafeine/. **Standard compositions**: www.coopathome.ch, www.produits.migros.ch, www.kinder.com, www.ferrero.fr, www.nu3.fr, www.ovomaltine.ch, www.nestle-shop.ch, www.merci.ch, www.nestle-cereals.com, www.i-dietetique.com, www.world-fr.openfoodfacts.org, www.leshop.ch, www.kelloggs.in, www.das-ist-drin.de, www.cailler.ch, www.lindt.ch, www.wikipedia.org, www.storck.ch. **Standard recipes**: www.swissmilk.ch, www.bettybossi.ch, www.cuisineaz.com, www.marmiton.org, www.750g.com, www.allrecipes.fr, www.fr.inshaker.com.*

Supplementary Table S2. Measurements of caffeine concentration in coffee and soft drinks.

Beverages	Description	Brands	Caffeine concentration (mg/100g)
Cola-based soft drinks		Pepsi®	7.7
		Pepsi Max®	10.0
		Coca Cola® (regular)	7.0
		Coca Cola Diet®	5.8
		Coca Cola Zero®	7.8
Iced teas		Ice Tea® lemon (Migros®)	4.1
		Lipton® lemon	3.1
		Nestea® lemon	3.6
		**Mean of iced teas**	**3.6**
Ristrettos	Self-made	Nespresso® ristretto 1	280.7
		Nespresso® ristretto 2	227.4
		**Mean of self-made ristrettos**	**254.1**
	Take-away/ restaurant/ vending machine	University hospital canteen 1 (Ticino)	227.5
		University hospital canteen 2 (Ticino)	225.2
		Starbucks® 1	406.2
		Starbucks® 2	396.2
		Selecta® 1	207.4
		Selecta® 2	217.9
		Professional Nespresso® 1	223.8
		Professional Nespresso® 2	237.9
		**Mean of take-away ristrettos**	**267.8**
		**Mean of ristrettos** (2/3 "self-made" and 1/3 "take-away")	**258.6**
Espressos	Powder-based (instant)	Instant Nescafé® Azera 1	40.0
		Instant Nescafé® Azera 2	37.2
		**Mean of powder-based espressos**	**38.6**
	Self-made	Nespresso® Livanto 1	93.7
		Nespresso® Livanto 2	94.6
		Nespresso® Capriccio 1	102.4
		Nespresso® Capriccio 2	94.1
		Nespresso® Volluto 1	81.4
		Nespresso® Volluto 2	77.5
		**Mean of self-made espressos**	**90.6**
	Take-away/ restaurant/ vending machine	University hospital canteen 1 (Ticino)	147.3
		University hospital canteen 2 (Ticino)	156.0
		Starbucks® 1	246.3
		Starbucks® 2	237.0
		Selecta® 1	183.6
		Selecta® 2	167.8

		Professional Nespresso® 1	137.6
		Professional Nespresso® 2	144.3
		**Mean of take-away espressos**	**177.5**
		**Mean of not powder-based espressos** (2/3 "self-made" and 1/3 "take-away")	**119.6**
Lungos	Powder-based (instant)	Instant Nescafé GOLD® 1	40.9
		Instant Nescafé GOLD® 2	40.1
		**Mean of powder-based lungos**	**40.5**
	Self-made	Nespresso® Envivo Lungo 1	64.4
		Nespresso® Envivo Lungo 2	53.9
		Nespresso® Fortissio Lungo 1	47.1
		Nespresso® Fortissio Lungo 2	55.5
		Nespresso® Vivalto Lungo 1	36.7
		Nespresso® Vivalto Lungo 2	45.2
		**Mean of self-made lungos**	**50.5**
	Take-away/ restaurant/ vending machine	University hospital canteen 1 (Ticino)	99.6
		University hospital canteen 2 (Ticino)	98.1
		Starbucks® 1	95.5
		Starbucks® 2	138.7
		Selecta® 1	119.4
		Selecta® 2	108.1
		Professional Nespresso® 1	72.4
		Professional Nespresso® 2	75.5
		**Mean of take-away lungos**	**100.9**
		**Mean of not powder-based lungos** (2/3 "self-made" and 1/3 "take-away")	**67.3**

Supplementary Table S3. Daily caffeine consumption per kg of body weight in the Swiss population (mg/kg/day) and percentage (%) of the population exceeding the recommendation of 5.7mg/kg/day.

Population characteristics		N	Weighted N	Weighted %	Weighted mean	SD	Weighted P5	Weighted P25	Weighted median	Weighted P75	Weighted P95	>5.7 mg/kg %
All	Entire population	2 057	4 627 878	100%	2.66	1.78	0.39	1.34	2.41	3.56	5.80	5.6%
Sex	Men	933	2 305 141	50%	2.66	1.76	0.42	1.37	2.43	3.54	5.60	4.5%
	Women	1124	2 322 737	50%	2.67	1.80	0.38	1.34	2.41	3.60	5.92	6.8%
Age group	18-34 years	563	1 306 178	28%	2.00	1.51	0.26	0.83	1.71	2.91	4.99	2.3%
	35-49 years	602	1 421 756	31%	2.77	1.81	0.41	1.44	2.46	3.67	6.12	6.4%
	50-64 years	554	1 250 918	27%	3.15	1.87	0.70	1.84	2.85	4.07	6.67	8.2%
	65-75 years	338	649 026	14%	2.82	1.62	0.54	1.80	2.64	3.64	5.79	5.5%
Language region	German-speaking	1 341	3 183 216	69%	2.83	1.87	0.38	1.42	2.56	3.81	6.27	7.1%
	French-speaking	502	1 187 738	26%	2.39	1.53	0.41	1.22	2.20	3.11	5.37	2.7%
	Italian-speaking	214	256 925	6%	1.93	1.23	0.37	0.99	1.75	2.73	4.02	0.6%
Nationality	Swiss	1 789	3 470 404	75%	2.66	1.78	0.39	1.41	2.42	3.55	5.79	5.5%
	Not Swiss	265	1 145 199	25%	2.66	1.79	0.37	1.23	2.41	3.55	5.92	5.8%
Education	Lower	286	620 712	13%	2.96	2.14	0.37	1.50	2.53	3.56	7.91	9.4%
	Middle	771	1 589 873	34%	2.44	1.61	0.36	1.16	2.20	3.36	5.56	4.5%
	Higher	997	2 405 018	52%	2.73	1.77	0.41	1.40	2.49	3.67	5.78	5.4%
Income	Lower	486	1 128 723	24%	2.69	1.93	0.35	1.26	2.45	3.46	6.12	6.8%
	Middle	516	1 095 517	24%	2.60	1.61	0.53	1.34	2.37	3.46	5.84	5.6%
	Higher	802	1 831 768	40%	2.69	1.73	0.41	1.40	2.42	3.65	5.60	4.7%
	No answer	250	559 595	12%	2.62	1.94	0.23	1.01	2.27	3.60	6.76	6.1%
Smoking status	Never smoked	1 072	2 307 169	50%	2.43	1.70	0.31	1.05	2.19	3.38	5.37	3.6%
	Ex-smokers	530	1 271 513	27%	2.70	1.60	0.49	1.57	2.45	3.55	5.79	5.8%
	Smokers	451	1 034 578	22%	3.13	2.05	0.69	1.80	2.73	3.96	7.12	9.7%
Weight status	Normal weight	1'166	2'625'518	57%	2.77	1.91	0.36	1.34	2.46	3.74	6.01	7.1%
	Overweight	629	1'422'231	31%	2.60	1.55	0.58	1.41	2.46	3.44	5.34	3.8%
	Obesity	262	580'130	13%	2.36	1.62	0.38	1.13	2.07	3.23	5.37	3.6%

N: number. SD: standard deviation. P: percentile.

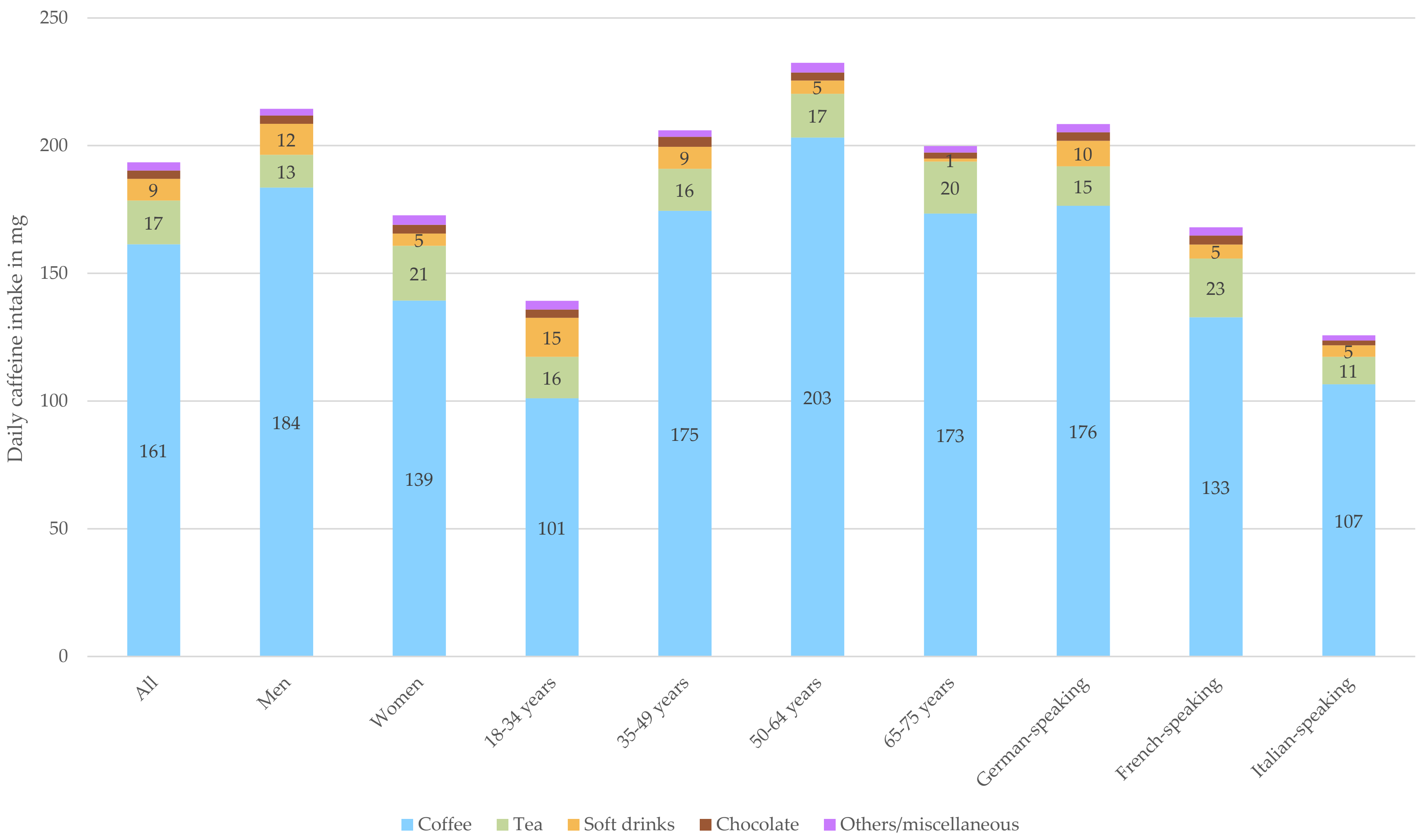

Supplementary Figure S2. Main food groups sources of caffeine (mg/day). *Labels on bars represent the intake of the specific food group.*

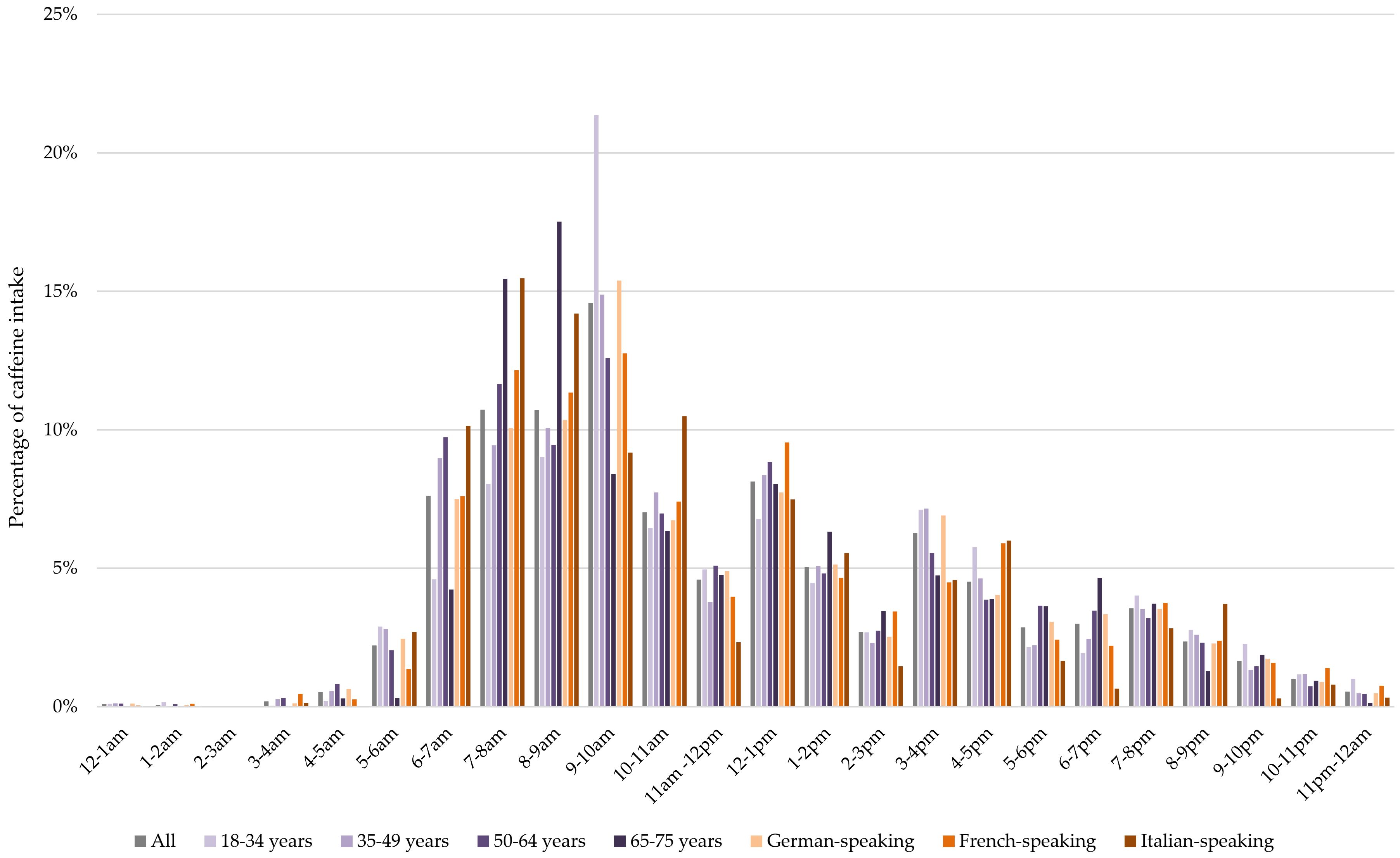

Supplementary Figure S3. Distribution of caffeine consumption per hour during the day (percentage/day).

Supplementary Table S4. Daily caffeine consumption in the Swiss population aged 18-64 years (mg/day) and percentage (%) of the population exceeding the recommendation of 400 mg.

Population characteristics		N	Weighted N	Weighted %	Weighted mean	SD	Weighted P5	Weighted P25	Weighted median	Weighted P75	Weighted P95	> 400mg %
All	Entire population	1 719	3 978 852	100%	190	132	28	92	166	259	428	6.9%
Sex	Men	765	1 960 574	49%	211	142	32	104	187	284	445	9.1%
	Women	954	2 018 277	51%	169	119	26	83	149	227	394	4.8%
Language region	German-speaking	1 108	2 700 260	68%	202	139	28	99	176	275	451	8.2%
	French-speaking	432	1 056 907	27%	170	115	29	86	147	232	400	4.9%
	Italian-speaking	179	221 685	6%	134	86	22	70	119	189	281	0.3%
Nationality	Swiss	1 464	2 870 497	72%	189	131	28	96	168	257	419	6.1%
	No Swiss	252	1 096 079	28%	191	135	31	83	163	266	445	9.0%
Education	Lower	208	489 881	12%	208	163	31	106	174	269	556	9.2%
	Middle	651	1 362 272	34%	172	121	25	78	155	238	408	5.4%
	Higher	857	2 114 423	53%	196	130	28	99	172	268	428	7.3%
Income	Lower	354	870 702	22%	188	140	29	85	163	250	481	7.3%
	Middle	414	900 718	23%	184	123	34	96	164	237	399	4.8%
	Higher	734	1 696 751	43%	194	128	28	98	173	266	416	6.4%
	No answer	214	498 405	13%	186	147	15	65	151	267	449	11.7%
Smoking status	Never smoker	907	1 994 752	50%	167	122	20	69	147	237	400	4.9%
	Ex-smokers	400	1 020 402	26%	197	117	39	110	172	269	416	6.9%
	Smokers	408	949 080	24%	229	155	46	131	199	285	519	10.8%
Weight status	Normal weight	1'022	2'342'545	59%	176	127	24	83	157	238	413	5.8%
	Overweight	495	1'173'135	29%	206	129	43	105	185	275	430	6.9%
	Obesity	202	463'172	12%	218	156	31	94	193	300	506	12.2%

N: number. SD: standard deviation. P: percentile.
